# Urinary VCAM-1 as a biomarker of lupus nephritis disease activity

**DOI:** 10.1186/1546-0096-12-S1-P108

**Published:** 2014-09-17

**Authors:** Eve Smith, Rachel Corkhill, Angela Midgley, Louise Watson, Caroline Jones, Stephen Marks, Kjell Tullus, Clarissa Pilkington, Michael W Beresford

**Affiliations:** 1 Institute of Translational Medicine, Women and Children's Health, University of Liverpool, UK; 2 Department of Paediatric Nephrology, Alder Hey Children's Hospital, Liverpool, UK; 3 Paediatric Nephrology Department, Great Ormond Street Hospital, London, UK; 4 Department of Paediatric Rheumatology, Great Ormond Street Hospital, London, UK

## Introduction

Up to 80% of children with Juvenile Systemic Lupus Erythematosus (JSLE) develop lupus nephritis (LN) (1), with the 5-year renal survival rate varying between 44-94% (2-4). Conventional markers of JSLE disease activity fail to adequately predict impending LN flares (5), with significant renal involvement (class III, IV or V LN) known to occur with low level proteinuria (6). Cross-sectional adult SLE studies have shown urinary vascular cell adhesion molecule-1 (VCAM-1) to be significantly higher in active LN than inactive LN or healthy controls, correlating with traditional markers of LN disease activity (7, 8).

## Objectives

To investigate the role of VCAM-1 as a urinary biomarker in JSLE.

## Methods

Urinary VCAM-1 concentrations were measured by ELISA (R&D Systems Ltd). The assay demonstrated 108-122% linearity of dilution, and 90-106% recovery using spike and retrieval techniques. Samples were diluted 1 in 80, and re-run at different dilutions where necessary. JSLE patients were classified as ‘JSLE active renal’ or ‘JSLE non-active renal’ based on the renal domain of the British Isles Lupus Assessment Group score (BILAG) (rBILAG A/B vs. D/E). Healthy children (HC), attending for non-inflammatory surgery were recruited as controls. Demographic, clinical and biomarker data were not normally distributed, and expressed as median values and interquartile ranges (IQR). Mann-Whitney U test was used when comparing between groups, and correlations utilized the Spearman rank test.

## Results

Sixty-seven patients participated in the study (50 JSLE patients and 17 healthy controls). JSLE patients had a median age of 16.5 years (range 10.07-21.91), and 36/50 (72%) were female. All JSLE patients had a median of 5 ACR classification criteria (IQR 4-7), with a median length of disease of 4.66 years (IQR 3.2-7.5). 23 (46%) JSLE patients were classed as JSLE active renal disease and 27 (54%) were JSLE non-active renal. Eleven (22%) JSLE patients had previously undergone a renal biopsy: Class IV LN (n=3), Class III (n=6) and Class II (n=2). The healthy controls had a median age of 12 years (range 4.0-16.0), with 5 being female (29%).

Urinary VCAM-1 levels were significantly higher in JSLE active renal patients (16.65 ng/mgCr [IQR 2.58-51.78]), versus non-active renal patients (2.3ng/mgCr [IQR 0.61-10.01], *p=0.002*) and HC's (2.4ng/mgCr [0.54-4.50], p=0.003). A statistically significant correlation was seen between VCAM-1 levels, C3 (*r= -0.38*, *p=0.009*) and urinary albumin-to-creatinine (UAUC) ratio (*r=0.49*, *p=0.001*).

## Conclusion

We have shown for the first time in children, that urinary VCAM-1 is able to identify patients with active renal lupus. Further assessment is required in prospective longitudinal studies.

## Disclosure of interest

None declared.

